# Bridging the Gap between Evidence and Practice for Adults with Medically Refractory Temporal Lobe Epilepsy: Is a Change in Funding Policy Needed to Stimulate a Shift in Practice?

**DOI:** 10.1155/2015/675071

**Published:** 2015-12-07

**Authors:** Alireza Mansouri, Abdulrahman Aldakkan, Magda J. Kosicka, Jean-Eric Tarride, Taufik A. Valiante

**Affiliations:** ^1^Division of Neurosurgery, University of Toronto, Toronto, ON, Canada; ^2^Toronto Western Hospital, University Health Network, Toronto, ON, Canada M5T 2S8; ^3^Department of Clinical Epidemiology and Biostatistics, McMaster University, Hamilton, ON, Canada L8P 1H1; ^4^Division of Neurosurgery, King Saud University, Riyadh, Saudi Arabia; ^5^Institute of Medical Sciences, University of Toronto, Toronto, ON, Canada; ^6^Division of Fundamental Neurobiology, Toronto Western Research Institute, Toronto Western Hospital, Toronto, ON, Canada M5T 2S8; ^7^Krembil Neuroscience Center, Toronto, Canada

## Abstract

*Objective*. Surgery for medically refractory epilepsy (MRE) in adults has been shown to be effective but underutilized. Comprehensive health economic evaluations of surgery compared with continued medical management are limited. Policy changes may be necessary to influence practice shift.* Methods*. A critical review of the literature on health economic analyses for adults with MRE was conducted. The MEDLINE, EMBASE, CENTRAL, CRD, and EconLit databases were searched using relevant subject headings and keywords pertaining to adults, epilepsy, and health economic evaluations. The screening was conducted independently and in duplicate.* Results*. Four studies were identified (1 Canadian, 2 American, and 1 French). Two were cost-utility analyses and 2 were cost-effectiveness evaluations. Only one was conducted after the effectiveness of surgery was established through a randomized trial. All suggested surgery to be favorable in the medium to long term (7-8 years and beyond). The reduction of medication use was the major cost-saving parameter in favor of surgery.* Conclusions*. Although updated evaluations that are more generalizable across settings are necessary, surgery appears to be a favorable option from a health economic perspective. Given the limited success of knowledge translation endeavours, funder-level policy changes such as quality-based purchasing may be necessary to induce a shift in practice.

## 1. Introduction

At an approximate global prevalence of 1%, epilepsy is among the most common serious neurological disorders worldwide [1]. Despite evidence in favor of the effectiveness of surgery for medically refractory epilepsy (MRE) [2–6], referral rates for evaluation of surgical candidacy are low [7–10]. Thus, many patients are maintained on ineffective and potentially harmful antiepileptic drugs (AEDs).

The economic impact of epilepsy should not be underestimated. The direct costs account for 25% of the societal economic burden [1, 11, 12]. In addition, there are indirect [12–14] and intangible costs [15]. Although seizure frequency has been shown to have a direct correlation with resource consumption [16], with seizure-free patients consuming 1/9th the resource, these figures should be balanced against the costs associated with presurgical evaluation, surgery, and its complications, along with accounting for possibility of ineffective surgery [17]. In implementing policy changes aimed at addressing possible societal welfare losses, funding organizations must balance effectiveness and costs associated with alternative interventions. Given the increasing demand for health care, rising costs, and the scarcity of resources, comprehensive health economic evaluations are necessary ingredients for guiding the decision-making process. Such economic evaluation, particularly since the landmark randomized trial suggesting the efficacy of surgery over best medical therapy [5], is however limited.

In this study, a systematic review of the literature was conducted to critically assess health economic evaluations specifically comparing surgery against continued AEDs in adults with medically refractory TLE. The overall findings have been evaluated in terms of their generalizability to other regions/health care systems. Furthermore, current obstacles to achieving efficient and equitable outcomes for MRE patients are considered. The merits of quality-based purchasing as a potential funder-level policy modification to overcome these obstacles are discussed.

## 2. Methods

### 2.1. Electronic Search

The MEDLINE, EMBASE, and Cochrane Library electronic databases were searched on February 14th, 2015. The Centre for Reviews and Dissemination (CRD) database (containing the Database of Abstracts of Reviews of Effects, Health Technology Assessment, and the NHS Economic Evaluation Database) along with the EconLit database was also searched. No limitations were placed on date of publication or language.

### 2.2. Search Strategy

The MEDLINE and EMBASE databases were searched separately, based on appropriate MeSH and EMTREE terms, respectively. Further details regarding the search strategy for these and other databases can be found in Appendix I in Supplementary Material available online at http://dx.doi.org/10.1155/2015/675071. Hand searching of the references for the selected articles was used to identify further relevant studies.

### 2.3. Title and Abstract Review

Titles and abstracts were reviewed independently and in duplicate (AM, AA); interobserver agreement was assessed using Cohen's Kappa score. Reviews, preliminary reports, protocols, and evaluations of alternative interventions for MRE (e.g., vagal nerve stimulators) were excluded.

### 2.4. Full-Text Review

The selected abstracts were reviewed independently and in duplicate (AM, AA) and full-texts were included if they pertained to adults with MRE in which a health economic evaluation comparing surgery and best medical therapy at the time was conducted; simple cost-analysis studies were excluded.

### 2.5. Data Extraction

Characteristics of the study with regard to design, population, and approach to health economic evaluation were extracted into a data extraction form that had been piloted and approved by the authors.

## 3. Results

**Figure 1:**
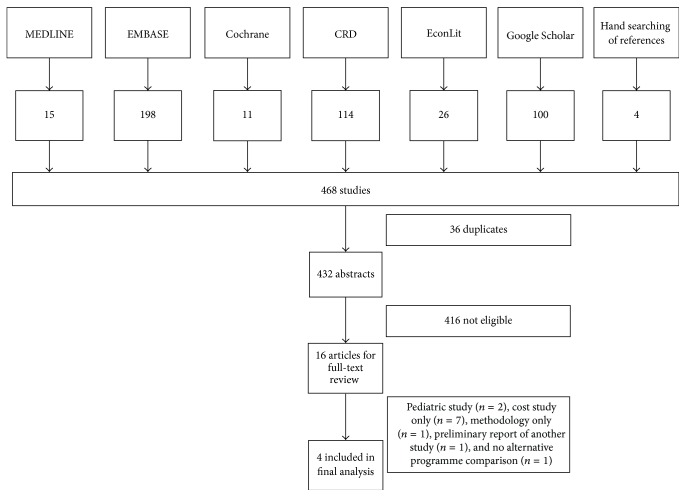
Flow diagram summarizing the results of the search strategy, followed by abstract and title screening.

Four studies were included (Figure 1); interobserver agreement at abstract (Kappa: 0.82) and full-text (Kappa: 0.89) was almost perfect. A summary of the characteristics of the included studies, along with the reason for exclusion of additional articles following full-text review, has been provided in Table 1. The specifics of the health economic evaluation in the included studies have been provided in Table 2.

### 3.1. Wiebe et al. (1995) [18]

In this Canadian study, a CEA from the provider perspective was undertaken. The primary effectiveness outcome was seizure-freedom status. Costs (1993 Canadian dollars) are comprised of AEDs, perioperative care, presurgical evaluation, and physician fees. These were obtained through surveying a small sample of the regional epilepsy population, assessment of the local patient cohort, hospital cost database, and physician reimbursement fees. A decision tree was constructed with transition probabilities obtained from the literature and verified by a panel of experts who also provided estimates of probabilities when not available. The model was applied to a hypothetical cohort of 100 patients in each arm and spanned a lifetime horizon (projected 35 years) discounted at 5%. A myriad of sensitivity analyses were performed; AEDs would be more cost-effective only if seizure-free rates in surgical cohort were <41% and >30% in AED cohort. Indirect costs were not addressed.

The cost per seizure-free patients was $895,119 and $142,419 in the medical and surgical cohorts, respectively. The upfront costs of surgery were recouped by the 9th year, after which costs continued to decline compared to medical cohort. The authors concluded that AEDs account for a large fraction of costs averted by surgery and although surgical costs are high, they are outweighed by those of medical management for TLE. Furthermore, the earlier the surgery is performed, the greater the savings are.

### 3.2. King et al. (1997) [19]

In this American study, a CUA from the societal perspective was conducted to compare presurgical evaluation and surgery against continued AEDs for MRE patients. The outcome was QALYs, derived from the literature. AED costs were assessed through a sample of 30 patients. Indirect costs were “explored” based on future earnings. Transition probabilities within the Markov model were obtained from direct data collection, published clinical trials, expert consensus, and clinical judgment. A lifetime horizon was considered, discounted at 5%.

Surgery was assumed to confer no survival benefit, but a mortality of 0.25% was assumed. From the simulations, average accumulated and discounted lifetime costs of AEDs were $8,000 USD for the surgically treated patients and $13,000 for medically treated patients. The marginal cost for evaluating and treating those with TLE was $29,800 USD (1994). The cost per QALY was $27,200, placing it well within the $50,000 threshold of what the authors considered reasonable for acceptable interventions.

### 3.3. Langfitt (1997) [20]

This American study was a CUA based on QALY difference in adults with medically refractory TLE. The provider perspective was considered and only direct medical costs were included; these were estimated from the local institution charges. Presurgical evaluation costs were obtained from 25 consecutive patients at the institution (1995 USD), whereas estimates of the complication costs were obtained from two patients who sustained intracranial hematomas. Follow-up costs were estimated from the literature and were a function of the extent of seizure control [21]. The authors argued against the validity of including indirect costs. All costs were discounted at 5%. A decision tree analysis was used, incorporating probabilities obtained from published values.

Base case analysis found an ICUR of $15,581 USD/QALY for surgery, below the upper ICUR threshold of $19,000 USD/QALY at the time (1995) [22]. In addition to various issues of generalizability with both the current analysis and that of King et al., the latter was based on an intent-to-treat analysis whereby all patients evaluated for surgical candidacy were assumed to belong to the surgical cohort; patients who are not candidates but continue on AEDs can erroneously increase costs in this cohort. Furthermore, Langfitt assumed that patients with Engel class I seizure status would not require any further follow-up, which would lower the AED and follow-up costs in the (likely more effective) surgical cohort. Sensitivity analyses suggested that efficiency of patient selection, the chance of being seizure-free after surgery, evaluation and follow-up costs, and exact estimate of QOL adjustments as a function of seizure frequency all affected the acceptability of the ICUR.

### 3.4. Picot et al. (2008) [23]

In this multicenter study based in France, a CEA was applied to a cohort of 280 patients with MRE (119: surgery, 161: AED). The primary effectiveness outcome was 1-year seizure-freedom rate. A Monte Carlo simulation of 1,000 patients based on the Markov transition model was used to expand the analysis to a lifetime horizon. The probability of seizure-freedom was based on study patients. Transition probabilities for the first four cycles were based on trial data while the rest were obtained from the literature. Mortality rates were assumed to be the age-equivalent population rates in France. Costs included inpatient/outpatient costs (2004 Euros), direct nonmedical costs (primarily transportation related, elicited from patients, 2003 Euros), and indirect costs (health capital approach, elicited from patients). The costs associated with patients undergoing intracranial EEG monitoring (27.7%) were also included. Costs were discounted at 3%.

Seizure-freedom rates at 1 year were 81.2% and 10.1% in the surgical and AED cohorts, respectively. These differences were stable beyond year 1. TLE was the diagnosis in ~85% of the surgical patients but only 58% of the medical cohort. Differences in costs were significantly in favor of the surgical cohort beyond 2 years, primarily attributed to reduction of AEDs. At the 5th postoperative year, the ICER for 1 seizure-free year for the surgical option was 1,900 Euros (direct costs only, 2004) and surgery became dominant at 7-8 years postoperatively. This benefit was delayed by ~1 year if a discount rate of 5% or if seizure-freedom rates from the literature were used. Significant variations were also noted when considering the extremes of surgical cost. Employment status was not significantly different. The authors concluded that if only direct costs are considered and effectiveness is defined as being seizure-free for 1 year, then the surgical option is cost-effective at ~7-8 years postoperatively. ICER thresholds were not used to determine cost-effectiveness.

## 4. Discussion

The effectiveness of surgery for medically refractory TLE has been established through various studies [5, 6, 28, 29]. In the current study, four health economic evaluations of surgery for MRE were identified through a systematic review, only one of which had been conducted following the RCT by Wiebe et al. [5]. All concluded surgery to be a favorable alternative to continued AEDs. However, the methodological details of these studies must be considered cautiously prior to applying their findings across various settings.

### 4.1. Critical Evaluation

In the Wiebe study, a hypothetical cohort (with limited description of patient characteristics such as MRE definition) was used and neither the quantity nor unit cost of many diagnostic investigations was provided. Modern day technology, costs, and practice protocol have changed since 1993. The definition of MRE used by King is no longer valid [30] and, similar to Wiebe, the cost data are likely outdated. In addition, the QALYs were based on health-related quality of life scales which had not been adjusted to reflect individual health state preferences for epilepsy patients. Furthermore, the QALYs at 1 year were summated and discounted to obtain lifetime values, assuming a constant relationship with time until death, which is not necessarily valid. Langfitt also used QALYs that had not been validated for individual health states. Furthermore, these were assumed to be dependent solely on seizure frequency. Similar to the above, cost values are likely outdated. The study by Picot was the only European study. Here an imbalance of baseline characteristics, with regard to proportion of TLE patients, was evident in the two treatment arms. Furthermore, costs were presented in aggregate format only. Together, these factors impact comparability across studies and generalizability to other settings.

Although the specific methodologies/assumptions of these studies may vary, the general conclusion is uniform. Cost analyses in other developing countries [24, 27, 31] have also demonstrated surgery to be cost-saving in the long term. Furthermore, two recent analyses conducted in Canada pertaining to children with MRE [9, 32] have also suggested the cost-effectiveness of surgery.

### 4.2. The Disconnect between Evidence and Practice

Despite the established effectiveness of surgery, referral rates for surgical evaluation continue to be low. In 2010, <750 individuals in Ontario (3.75% of the potential 20,000 surgical candidates) were assessed for candidacy [33]. The estimated wait-time from first seizure to surgery can be as long as 22 years [34–37]. Ontario is not unique for this “treatment gap,” which is reflective of the state of epilepsy care in Canada, North America, and much of the rest of the world [7]. The medical community's skepticism toward surgery [38, 39] and variable definitions of MRE [39, 40] have contributed to these statistics. However, despite class I evidence in favor of early referral for surgical assessment, a change in practice has not been observed [41].

A delay in the comprehensive management of patients with epilepsy has various negative biopsychosocial and ethical repercussions [42–46]. The Ontario health technology assessment committee states that patients with MRE should be considered as surgical candidates unless proven otherwise [42]. The American Academy of Neurology has recommended that patients are reassessed for surgical candidacy every 3 years as part of quality-care indicators [47]. Given such positions and the limited success of knowledge translation endeavours, consideration of funder-level policy changes to promote a shift in practice may be warranted. The limitation of resources such as specially trained health care professionals and appropriate diagnostic tools is certainly a contributing factor. However, this scarcity expands across the entire economic landscape of medicine and simply increasing available resources will not be a sustainable solution. As an alternative funder-level policy adaptation, quality-based purchasing (QBP) is an option for ensuring delivery of high quality care [48]. In the following section, the strengths and limitations of various strategies toward achieving QBP are discussed.

### 4.3. Quality-Based Purchasing

In the principal-agent framework that describes the relationship between the funder (principal) and providers (agents) [49], the principal strives to provide necessary information and incentives to align the agent toward a unified goal: quality care. The information can be guidelines/performance targets while incentives can be financial/nonfinancial. Physician personality traits (e.g., personal motivation for improvement and altruism) are strong nonfinancial factors and should be explored [50, 51]. Related to physician personality, some suggest that providing financial incentives based on patient satisfaction surveys rather than productivity goals may be better received [52]. While reasonable, these measures are potentially subjective and therefore difficult to quantify. Consideration of options for financial incentives based on quality care criteria is discussed below.

### 4.4. Parameters to Consider

Any incentive scheme is likely associated with positives and negatives; careful consideration of several factors is necessary. Baseline characteristics of physicians and structure of practice are influential as habits and established practice patterns are harder to modify [53]. The perception of the target group on the attainability of the quality index matters; physicians may be more open to measures based on the structure/process of care delivery (e.g., appropriate timely referrals) compared to outcome measures (e.g., number of seizure-free patients) [54]. The decision to impose penalties or incentives is paramount; while it is conceivable that the former is more likely to be influential, the repercussions must also be considered.

### 4.5. Penalty-Based Schemes

The imposition of penalty-based reforms in Germany (1993) [55] and British Columbia (early 1990s) [56] targeting the rising expenditures on medications resulted in a swift change in practice and reduction of costs. However, dissatisfaction was an issue in both cases. Imposition of funding penalties to the restricted setting of physicians/clinics caring for epilepsy patients based on well-defined referral criteria may increase referral rates with minimal adverse effects on other interventions. Considering the myriad of evidence in favor of the effectiveness (from health and economics perspective) and the established quality-based standards, this approach may be justified. However, this represents a rather antagonistic approach that decreases overall satisfaction and hampers the collaborative approach to patient care. Furthermore, physicians may choose against enrolling epilepsy patients based on concerns of being penalized for inappropriate care.

### 4.6. Pay-for-Performance (PFP)

Incentives for performance promote a more positive approach and ideally improve quality care [57]. However, success has been limited. In Ontario (2002), a PFP strategy was initiated to optimize several preventative care services. Incentives (as high as 10% of the physicians' gross annual income) pertained to both the initiations of contact with eligible patients and of achieving cumulative preventative care targets [58]. Only modest increases were noted, likely attributable to the inability to affect patient demand, the amount of the incentive being too small, and the range of services affected by these incentives being too broad and confusing [58]. The UK NHS implemented a similar strategy though incentives were higher and yet improvement in quality of care was not observed for all intended programs; some areas not covered by the incentives declined further [59]. Elements from these and other failed initiatives provide useful insight [60, 61]. It is clear that the type of bonus matters and the amount must make the endeavour worthwhile [62, 63]. The guidelines should be simple to understand and implement [63]. The potential for “cream skimming” is a concern [60]. Furthermore, strategies are necessary to ensure care in other areas is not compromised [63]. The selection of appropriate performance targets and their appropriate measurement would be a challenge.

In Ontario, a provincial strategy for improving epilepsy care was proposed in 2011 to expand on infrastructure and to promote regionalization of care [64]. District epilepsy centers serve as nodes of contact between community physicians and regional epilepsy centers of excellence (ECEs). These districts provide initial diagnostic evaluation, connect patients with advocacy groups, provide recommendations, and coordinate with ECEs regarding further assessment and care. Such a network provides the ideal setting for the implementation of a comprehensive yet simple PFP strategy. A similar framework (e.g., accountable care organizations) can be implemented in other healthcare funding models as well [65]. The possibility for establishing and strengthening existing collaborations with patient advocacy groups within the network to increase patient awareness regarding available resources is one key benefit. Further, districts provide the ideal hub for the dissemination of evidence, guidelines, and expectations to community physicians. Reimbursing community physicians and ECE epileptologists/neurosurgeons set amounts for the* collective* management of the region's epilepsy patients recognizes all stakeholders, increasing buy-in and thus collaboration. Incentives directed at community physicians (for accepting new patients and referring MRE patients according to guidelines) and ECE physicians (for timely assessment and provision of care) can improve patient flow. To avoid “cream skimming” for patients who are good surgical candidates, a capitation approach, funding based on number and variety of epilepsy patients enrolled, would be necessary. To ensure continuity, guidelines for discharging seizure-free patients should be provided. Although the ideal size of the bonus would be challenging to determine, it must outweigh the opportunity cost for physicians. Frequent evaluation of the results and long-term financial implications of this policy change are necessary as well.

### 4.7. Future Directions

There is a need for updated health economic evaluations incorporating modern day costs. Ideally these would be conducted through RCTs and would be multinational to increase generalizability, particularly given the large variations in global costs of AEDs and surgery [20, 25, 31]. Although indirect costs are controversial and difficult to quantify, a systematic approach toward assessing them in epilepsy patients would be worthwhile. Furthermore, all of the studies reviewed pertained to established epilepsy centers; none considered the capital costs of establishing epilepsy centers/expanding the existing infrastructure. This is relevant considering the potential need for expansion of ECEs in anticipation of the potentially increased streamlining of referrals.

### 4.8. Conclusions

The expanding body of evidence and the uniform conclusions of the analyses reviewed suggest that, for MRE patients, surgery is likely to be in fact cost-saving over the long term [18, 23]. Despite published guidelines, referral rates of MRE patients for surgical evaluation continue to be low on a global level. Therefore, funding reforms may need to be considered to stimulate change. Ultimately reforms in funding alone are not sufficient but until large-scale shifts in the medical culture are implemented, this may be a worthwhile alternative [66].

## Supplementary Material

Summary of search strategy employed for systematic review.

## Figures and Tables

**Table:** (a) Studies that were included in final analysis

First author/year	Home nation	Study population	Source of costs	Source of outcomes	Funding	Recommendations
Wiebe/1995 [18]	Canada	MRE adults with presumed TLE (hypothetical cohort of 100 patients in each alternative option)	*Preliminary resource consumption* Survey of 33 representative local patients *Confirmation of resource use* Expert panel *Perioperative costs* Cohort of 30 consecutive local patients (1993 Cdn) *Physician costs* Provincial fee schedule (1992 Cdn)	*Clinical outcome probability estimates*, Literature search, local experience, and expert panel	N/A	Surgery is cost-effective $895,119/seizure-free patient with BMT versus $142,419/seizure-free patient with surgery Surgery dominates BMT at around 8-9 years

King/1997 [19]	USA	51 MRE adults with TLE	*Hospital costs* Cost/charge ratios from local finance department (1994 USD) *Outpatient investigations/physician costs* Medicare fee schedule (1994 USD) *AED cost* Local bulk acquisition cost (1994 USD)	*One-year seizure status*, Cohort of 51 local patients *Postoperative mortality*, Review of the literature and local data *Nonsurgical mortality*, Review of the literature *QALYs*, Review of the literature	N/A	ATL is preferred for MRE (ICUR^*∗∗*^ of surgery = $27,200/QALY)

Langfitt/1997 [20]	USA	Hypothetical cohort of MRE adults with TLE	*Medical services* Local hospital and providers plus review of the literature (1995 USD) *Surgical evaluation* Based on select cohort of 25 local patients (1991–1993 USD) *Surgical complication costs* Based on postoperative hematoma costs of 2 patients in larger operative cohort of 150 patients (1991–1993 USD) *Follow-up costs* Lifetime estimate, using local unit costs (year not clear) *AED costs* Hospital pharmacy costs based on average AED dosage (year not clear)	*Clinical event probabilities*, Review of the literature *QALY*, Review of the literature	N/A	ATL is preferred for MRE (MCUR^*∗∗*^ of surgery = $15,581/QALY)

Picot/2008 [23]	France	280 adults with MRE thought to be surgical candidates (not necessarily TLE)	*Hospital costs* Published fees (2004 Euros) *Outpatient costs* Published professional fees (2004 Euros) *Direct, nonmedical costs* Estimated from mode of transportation, distances, and transport fees (2003 Euros), elicited from patients *Indirect costs* Working days lost, elicited from patients	*Seizure freedom rates*, Review of outcomes for 280 patients in study *Transition probabilities*, Review of the literature *Mortality rates*, General population data *Quality of life*, Questionnaires administered to patients in study	National PHRC (1998) and Pfizer	Surgery is cost-effective in medium-term projections (productivity not considered) ICER of surgery at 5 years: ~1,900 Euros/seizure-free year Surgery is cost-effective at around 7-8 years postoperatively (ICER becomes 0, direct costs only)

**Table:** (b) Studies that were NOT included in final analysis

First author/year	Home nation	Title	Journal	Reason for exclusion	Main conclusions
Rao/2000 [24]	India	Is Epilepsy Surgery Possible in Countries with Limited Resources?	Epilepsia	Isolated cost analysis	(i) Surgery for MRE is feasible in developing nations (ii) Surgery is cost-effective

Platt/2002 [25]	USA	A Comparison of Surgical and Medical Costs for Refractory Epilepsy	Epilepsia	This was a cost analysis to assess the impact of incorporating direct and indirect costs	(i) Surgery is cost-effective (ii) Reduction of direct costs occurs in the long term (>10 years) (iii) Income gains more significant to society than payers; therefore, societal perspective is necessary

Picot/2004 [26]	France	Cost-Effectiveness of Epilepsy Surgery in a Cohort of Patients with Medically Intractable Partial Epilepsy—Preliminary Results	Revue Neurologique	Preliminary report of longer-term study already included in this review	Surgery was cost-effective at around 7-8 years after intervention

Chen/2014 [27]	China	Surgery: A Cost-Effective Option for Drug-Resistant Epilepsy in China	World Neurosurgery	Review of cost studies pertaining to surgery for MRE in China	Surgery is a cost-effective option for patients not responding to medications

MRE, medically refractory epilepsy; TLE, temporal lobe epilepsy, QALY, quality-adjusted life year, and AED, antiepileptic drug; BMT: best medical therapy; ICER, incremental cost-effectiveness ratio, MCER, marginal cost-effectiveness ratio, and ICUR, incremental cost-utility ratio; ATL, anterior temporal lobectomy.

^*∗∗*^Authors reported ICER in original publication.

**Table 2:** Specific components of health economic evaluation conducted in selected studies.

First author	Type of economic evaluation	Outcome measure	Perspective	Modeling	Time horizon (years)	Discounting (rates in %)
Wiebe [18]	Intent-to-treat, CEA	Seizure freedom rate overall	Provider	Decision analysis modeling	Lifetime (35 years)	5

King [19]	Intent-to-treat, CUA^*∗*^	QALY	Societal	Markov state transition model	Lifetime	5

Langfitt [20]	CUA^*∗*^	QALY	Provider	Decision analysis modeling	Lifetime	5

Picot [23]	CEA alongside clinical study (280 patients total)	Seizure-freedom rate at 1 year	Societal	Monte Carlo simulation based on Markov transition model	Lifetime	3

^*∗*^Although referred to as a CEA, this was technically a CUA.
