# Supplementary material for: Bridging the Gap between Evidence and Practice for Adults with Medically Refractory Temporal Lobe Epilepsy: Is a Change in Funding Policy Needed to Stimulate a Shift in Practice?
Source: Epilepsy Res Treat. 2015 Dec 7;2015:675071. doi: 10.1155/2015/675071 (PMC4685103; doi:10.1155/2015/675071)
Supplement: Supplementary file 1 — Summary of search strategy employed for systematic review. [file 675071.f1.doc]

Database: Ovid MEDLINE(R) <1946 to February Week 2 2015>

Search Strategy:

--------------------------------------------------------------------------------

1 exp Cost-Benefit Analysis/ (61238)

2 cost benefit.mp. (64681)

3 cost utility.mp. (2350)

4 cost effectiveness.mp. (33298)

5 exp Temporal Lobe/ (27242)

6 exp Epilepsy, Temporal Lobe/ (10339)

7 exp Epilepsy/ (130565)

8 5 and 7 (4688)

9 1 or 2 or 3 or 4 (77492)

10 6 or 8 (12499)

11 9 and 10 (15)

***************************

Database: Embase Classic+Embase <1947 to 2015 Week 07>

Search Strategy:

--------------------------------------------------------------------------------

1 temporal lobe.mp. or exp temporal lobe/ (81733)

2 exp epilepsy/ or epilepsy.mp. (210097)

3 exp temporal lobe epilepsy/ or exp lateral temporal lobe epilepsy/ or exp intractable epilepsy/ or exp mesial temporal lobe epilepsy/ (21808)

4 1 and 2 (25045)

5 3 or 4 (29599)

6 exp "cost benefit analysis"/ (66683)

7 exp "cost effectiveness analysis"/ (103630)

8 exp "cost utility analysis"/ (5868)

9 exp health economics/ (642950)

10 6 or 7 or 8 or 9 (642950)

11 5 and 10 (199)

12 remove duplicates from 11 (198)

***************************

Database: Cochrane February 2015

Title or abstract or keyword search for:

1. Mesial temporal lobe epilep* (53)
2. Hippocamp* (827)
3. Temporal lobe* (892)
4. Cost benefit* (20648)
5. Cost effective* (22738)
6. 1 OR 2 OR 3 (1533)
7. 4 OR 5 (30646)
8. 6 and 7 (11)

CRD database (114):

“epilepsy” AND “cost” within any field were combined

EconLit (26):

A query of any sources containing the subject headings “epilepsy” or “seizure” or the term “epilepsy” or “seizure” within the abstract was conducted
